# Chitosan Nanoparticles Unlock the Antioxidant Potential of Epigallocatechin Gallate in Pancreatic and Hepatic Cancer Cell Models

**DOI:** 10.1021/acsomega.6c02803

**Published:** 2026-05-07

**Authors:** Annalisa Bianco, Paolo Pellegrino, Mariafrancesca Cascione, Riccardo Dicorato, Livia Giotta, Lorenzo Vincenti, Isabella Farella, Loris Rizzello, Rosaria Rinaldi, Valeria De Matteis

**Affiliations:** † Department of Experimental Medicine, 18976 University of Salento, Via Monteroni, 73100 Lecce, Italy; ‡ Institute for Microelectronics and Microsystems (IMM), CNR, Via Monteroni, 73100 Lecce, Italy; § Department of Mathematics and Physics “E. De Giorgi”, University of Salento, Via Monteroni, 73100 Lecce, Italy; ∥ Istituto Italiano di Tecnologia (IIT), Via Barsanti, Arnesano, 73010 Lecce, Italy; ⊥ Department of Biological and Environmental Sciences and Technologies, University of Salento, S.P. Lecce-Monteroni, 73100 Lecce, Italy; # Department of Pharmaceutical Sciences (DISFARM), 9304 University of Milan, Via Luigi Mangiagalli, 25, 20133 Milan, Italy; ¶ Infection Dynamics Laboratory, National Institute for Molecular Genetics (INGM), Milan, Via Francesco Sforza, 35, 20122 Milano, Italy

## Abstract

Epigallocatechin gallate (EGCG), the predominant catechin in green tea, displays strong antioxidant and cytoprotective properties; however, its poor stability and limited oral bioavailability significantly restrict its therapeutic potential, prompting the need for new therapeutic strategies. Here, we reported synthesis and physicochemical characterization of chitosan nanoparticles (Ch-NPs) encapsulating EGCG (Ch-EGCG-NPs) by the ionic gelation method. The Ch-EGCG-NPs displayed tunable particle size (184–236 nm), narrow polydispersity, and positive zeta potential, supporting colloidal stability and efficient EGCG loading. NPs uptake was followed by confocal microscopy and flow cytometry, using FITC-labeled Ch-NPs demonstrating efficient internalization in pancreatic and hepatic cancer cell models, i.e HepG2 and PANC-1 respectively. In vitro assays revealed that Ch-EGCG-NPs preserved cell viability under hydrogen peroxide–induced oxidative stress and significantly modulated intracellular ROS levels. The oxidative treatment induced a 1.89-fold increase in ROS production compared to the control (corresponding to an 89% up-regulation), representing the largest percentage change observed. Conversely, Ch-EGCG-NPs markedly reduced ROS accumulation compared to free EGCG, lowering ROS levels from 2.24 to 1.06 (≈52.7% reduction) and from 2.24 to 0.66 (≈70.5% reduction), with reductions ranging from 52% to 70.5% in PANC-1 cells and up to 60.3% in HepG2 cells.

## Graphical abstract

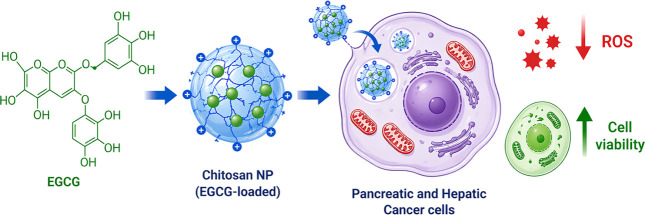

## 1 Introduction

Epigallocatechin gallate (EGCG) is the dominant catechin in green tea (*Camellia sinensis*), which is the most abundant polyphenol. Green tea also contains other structurally related catechins, including (+)-catechin, (−)-epicatechin (EC), and (−)-epicatechin-3-gallate (ECG).^1^ Among these compounds, EGCG has attracted particular attention due to its strong anticancer,^2,3^ antioxidant,^4^ and antimicrobial activities. Its antioxidant effects are largely attributed to its ability to scavenge reactive oxygen species (ROS) and regulate peroxidase activity, thereby mitigating oxidative stress and its associated cellular damage. These properties have positioned EGCG as a promising bioactive compound for potential applications in the prevention of oxidative stress–related diseases.^5–8^ However, the practical application of EGCG is limited by its poor oral bioavailability, typically below 2–5%.^9,10^ Tea catechins generally show low bioavailability and they undergo rapid systemic clearance, resulting in a short plasma half-life.^11^ Although ingestion of tea catechins has been shown to increase the antioxidant capacity of human plasma transiently, their concentrations decline rapidly after administration.^12^ This limitation is primarily attributed to their chemical instability and susceptibility to enzymatic and microbial degradation in the gastrointestinal tract. In addition, catechins are chemically unstable in solution, tending to degrade through oxidative reactions^13^ and lose their original structure. This degradation process is affected by environmental factors such as pH, temperature, and exposure to UV light,^14–16^ accelerating catechin breakdown and reducing their effectiveness. Therefore, strategies to improve both the stability and bioavailability of EGCG are key factors to advance its use in the food and health industries, where stability during processing and storage is essential.^17^ In this context, Ch-NPs emerge as a highly promising delivery platform, combining biocompatibility and biodegradability with the ability to protect and stabilize sensitive bioactive molecules, while overcoming the limitations and concerns associated with inorganic materials, particularly regarding toxicity and biodistribution.^18–20^

When used to encapsulate EGCG, Ch-NPs can protect the compound from gastrointestinal degradation, improve its bioavailability, and potentiate its antioxidant activity. The work presented here reported the synthesis of bare Ch-NPs and EGCG-loaded Ch-NPs by a modified ionic gelation method. The novel nanostructures were thoroughly characterized to evaluate their physicochemical properties following the encapsulation of EGCG into Ch-NPs. Cytotoxicity of bare and EGCG-loaded Ch-NPs was evaluated in hepatic and pancreatic cancer cell line models (HepG2 and PANC-1, respectively) in terms of viability and oxidative stress. These two cell lines were selected based on compelling evidence that pancreatic and hepatic cancers are characterized by sustained oxidative stress and extensive redox metabolic reprogramming.^21,22^ Yet, therapeutic strategies capable of selectively targeting this redox imbalance are still limited. Moreover, high-resolution confocal microscopy was employed to examine NPs’ internalization, intracellular distribution, and the influence of EGCG-loaded Ch-NPs on cellular morphology, including actin organization and variation in mitochondrial membrane potential-dependent signal. The present study demonstrates that Ch-NPs not only preserve the physicochemical integrity of the EGCG but also markedly enhance its cellular uptake and cytoprotective efficacy under oxidative stress conditions. These findings position EGCG-loaded Ch-NPs as a promising platform for antioxidant-based therapeutic strategies.

## 2 Materials and Methods

### 2.1 Synthesis of Ch-NPs, Loaded with ECGC, by Ionic Gelation Method

The Ch-NPs were obtained using low molecular weight chitosan that was dissolved in 1% (v/v) acetic acid, prepared with sterile, nuclease-free water, at a concentration of 1.0 mg/mL, under continuous stirring at 1000 rpm on a hot plate, maintained at ∼80 °C for at least 24 h. Simultaneously, TPP powder was dissolved in sterile, nuclease-free water at a concentration of 1.0 mg/mL under continuous stirring (∼500 rpm, 5 min). Lastly, EGCG was dissolved in nuclease-free water at a concentration of 2 mg/mL. All solutions were filtered through a 0.22 μm syringe filter (Millipore, USA), to eliminate impurities and undissolved particles and to further sterilize solutions (Figure 1a).

**Figure 1:**
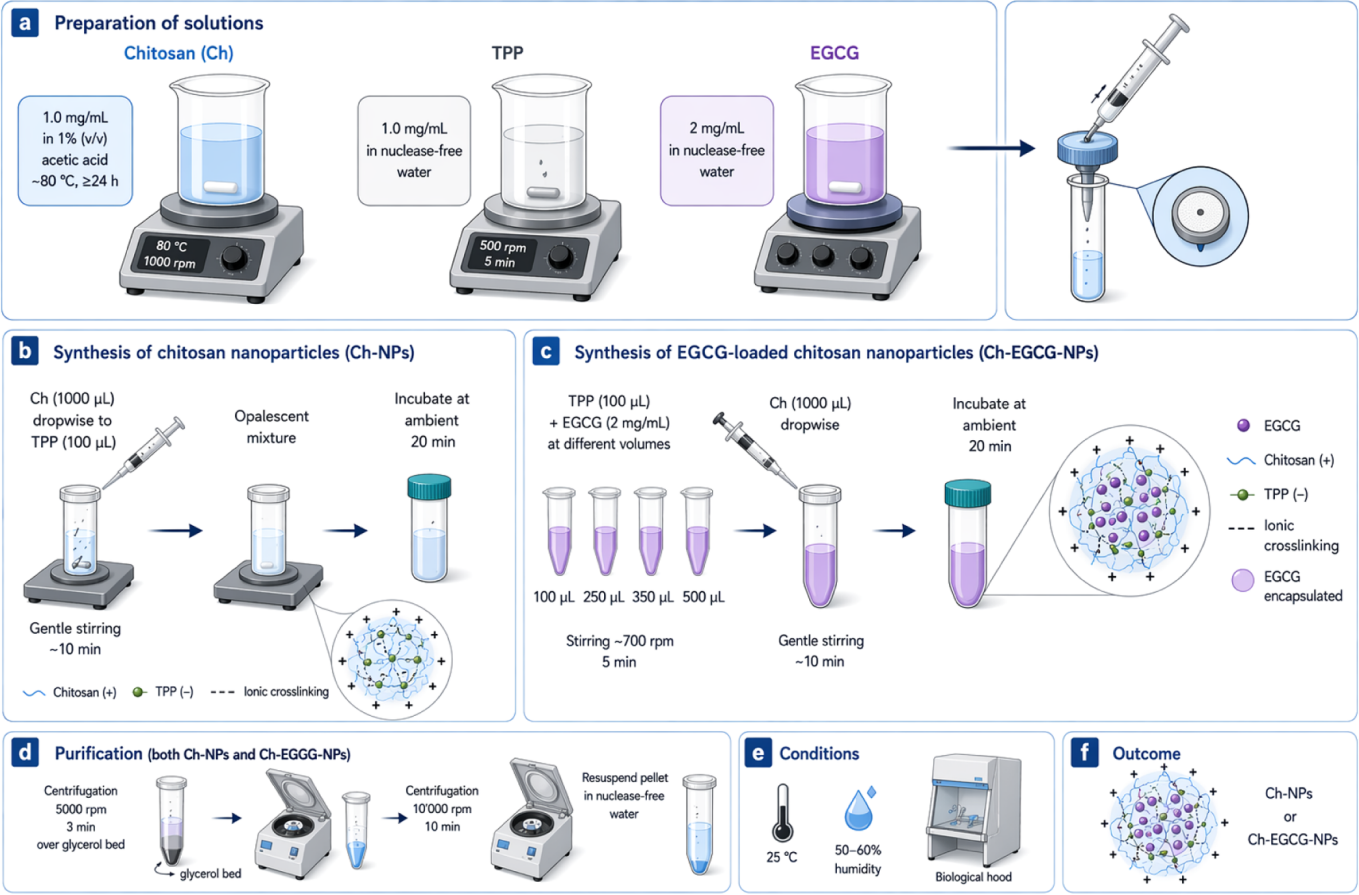
Schematic representation of the synthetic procedure for bare Ch-NPs and EGCG-loaded Ch-NPs.

The synthesis of the chitosan-based NPs was carried out by adding drop-by-drop, 1000 μL of Ch solution (1.0 mg/mL) to 100 μL of 1.0 mg/mL TPP, under gentle magnetic stirring for about 10 min, until the mixed solution appeared opalescent. Then, the stirring was stopped, and the solution was left at ambient for 20 min to allow the NP assembly (Figure 1b).

Similarly, to obtain EGCG-loaded Ch-NPs, 100 μL of 1.0 mg/mL TPP was first mixed with EGCG solutions at a concentration of 2 mg/mL, with increasing volumes (100 μL, 250 μL, 350 μL, and 500 μL) by stirring at about 700 rpm for 5 min to permit an effective mixing of the two solutes. This allowed efficient encapsulation of EGCG via electrostatic interactions during nanoparticle formation. Successively, 1000 μL of CS solution (1.0 mg/mL) was added dropwise under gentle stirring. The resulting suspension was kept stirring for 10 min at room temperature and further incubated for 20 min to promote nanoparticle self-assembly and stabilization. Nanoparticles were collected by sequential centrifugation: first at 5000 rpm for 3 min over a glycerol bed, then at 10′000 rpm for 10 min, after which the pellet was resuspended in nuclease-free water (Figure 1c). The Ch-NPs and ECGC-loaded NPs were synthesized under ambient conditions (25 °C, 50–60% humidity) in a biological hood and purified using the same procedure (Figure 1d). The resulting NPs (Figure 1d) were then characterized.

### 2.2 FITC Labeling of Ch-NPs and Conjugation Efficiency Analysis

Nanoparticles were purified to remove residual reactants and then resuspended in a 5 μM FITC solution. The conjugation reaction was carried out under gentle conditions, in the dark at 4 °C for 12 h, to preserve both fluorescence and nanoparticle integrity. Following incubation, FITC-conjugated nanoparticles were washed and centrifuged at least three times to eliminate unbound FITC and reaction byproducts. The labeled nanoparticles were stored in a protected environment from light until use.

FITC conjugation efficiency was determined indirectly by measuring the fluorescence of the total FITC added to the reaction and subtracting the fluorescence of unbound dye remaining in the supernatant after centrifugation. Nanoparticles were separated from free FITC by centrifugation at 10′000 rpm for 10 min. Fluorescence intensity was quantified using a BioTek Gen5Microplate Reader (Agilent Technologies, USA) with excitation at 495 nm and emission at 520 nm. A standard curve of free FITC in the same solvent was used to calculate the amount of conjugated dye. The conjugation efficiency was calculated using the following eq 1

1 $$FITC\;conjugation\;efficiency\left(\%\right)=\left(\frac{\left(F_{total}-F_{supernatant}\right)}{F_{total}}\right)\times 100$$

where *F*_total_ is the fluorescence of the initial FITC solution (before reaction), and *F*_supernatant_ is the fluorescence of the unbound FITC in the supernatant. All samples were measured in triplicate.

### 2.3 Characterization of Bare and EGCG-Loaded Ch-NPs

#### 2.3.1 Dynamic Light Scattering (DLS)

The physicochemical properties of the nanoparticle formulations, including Chitosan NPs, and EGCG-loaded Ch-NPs, were characterized using a Zetasizer Nano-ZS system (ZEN3600, Malvern Instruments Ltd., Malvern, UK) (Figure 1e). Parameters assessed included hydrodynamic diameter (Z-average size), polydispersity index (PDI), and Zeta-potential (ζ). Measurements were performed at 25 °C and pH 7.0 in aqueous solution, following a 120 s pre-equilibration period. The instrument, equipped with a 4.0 mW HeNe laser operating at 633 nm and a fixed scattering angle of 173°, was used to analyze 500 particles per sample. Size distributions were fitted using a normal Gaussian function to ensure statistical representativeness. Each result represents the average of five 60 s measurements. For each measurement, 10 μL of either pure Ch-NPs or EGCG-loaded Ch-NPs solution was diluted in 990 μL of nuclease-free water, and the suspension was transferred to a 1 mL polystyrene cuvette for analysis. The samples were analyzed in triplicate to ensure reproducibility.

#### 2.3.2 Transmission Electron Microscopy (TEM)

Following the synthesis and successive sequential purification, the Ch-NPs were resuspended in ultrapure water for morphological analysis. More in detail, the nanoparticle suspensions were diluted 1:50 in ultrapure water and deposited on carbon-coated copper grids for transmission electron microscopy (TEM). The samples were dried out under a chemical fume hood for at least 12 h. The nanoparticle morphology was assessed by using a JEOL M-1011 TEM microscope, operated at 100 kV, and equipped with a 7.1-megapixel CCD camera (Orius SC1000, Gatan, Pleasanton, CA, USA). Image acquisition and analysis were conducted using Gatan Digital Micrograph (DM) software. Samples were prepared by diluting 1 μL of Ch-NP or Ch-ECGC-NP solution into 9 μL of nuclease-free water; the NPs solutions were deposited on carbon-coated copper grids, blotted and air-dried, and incubated for at least 12 h before being examined (Figure 1e).

#### 2.3.3 Atomic Force Microscopy (AFM)

The morphology of Ch-NPs, diluted 1:50 in ultrapure water and deposited on freshly cleaved MICA, was examined using an atomic force microscope (AFM) (NTEGRA, NT-MDT Spectrum Instruments, Moscow, Russia) equipped with spectroscopy and nanolithography modules. Measurements were performed under ambient conditions (∼20 °C, 50–60% relative humidity). High-resolution topographic imaging was carried out in semicontact error mode by using NSG01 probes (NT-MDT Spectrum Instruments) with a nominal tip radius of ∼6 nm, a resonant frequency of 150 kHz, and a spring constant of 5.1 N/m. Data were acquired at a set point current of ∼5.0 nA, a gain of 0.80, and a scan rate of 0.30 Hz.

Two signal channels were collected simultaneously: (i) SensHeight, providing quantitative surface topography, and (ii) Magnitude, recording the amplitude error signal from the feedback loop. In semicontact error mode, the Magnitude signal reflects transient deviations in cantilever oscillation relative to the set point, arising from the finite response time of the feedback system. Expressed in nanoamperes (nA), this channel provides complementary morphological contrast, enabling the detection of fine structural features that may not be fully resolved in SensHeight data. By comparing both channels, it is possible to improve the accuracy and reliability of surface characterization.^23^

#### 2.3.4 Fourier Transform Infrared (FTIR) Spectroscopy

Fourier transform infrared (FTIR) spectroscopy was employed to confirm the presence of TPP and chitosan in the nanoparticles. The spectra were analyzed for characteristic peaks corresponding to the functional groups of both TPP and chitosan, confirming their successful incorporation into the nanoparticle’s matrix. FTIR spectra were acquired in the mid-infrared region using a Spectrum One FTIR spectrophotometer (PerkinElmer, Waltham, MA, USA) equipped with an attenuated total reflectance (ATR) accessory featuring a three-bounce diamond microprism (4 mm diameter) as the internal reflection element (IRE). Approximately 2 μL of nanoparticle suspension was deposited onto the ATR crystal; after solvent evaporation, spectra were recorded at a resolution of 4 cm^–1^. For each sample, 16 interferograms were collected and averaged to improve the signal-to-noise ratio. Background spectra were recorded by using the clean diamond microprism prior to sample measurement.

#### 2.3.5 Evaluation of ECGC Encapsulation Efficiency Antioxidant Activity Measurements

The antioxidant capacity of the samples was evaluated through the well-established ABTS decolorization assay using EGCG as a standard. The ABTS decolorization assay assesses the ability of antioxidants to quench the ABTS^•+^ radical cation, a chromophore with characteristic absorbance peaks at 645, 734, and 815 nm in an aqueous environment. Antioxidants reduce ABTS^•+^, regenerating the neutral form, which is colorless. The resulting absorbance drop (Δ*A*) is proportional to the overall antioxidant capacity of the sample, which in turn depends on both concentration and specific antioxidant capacity of the antioxidant(s) present in the solution. Depending on the antioxidant compound employed as standard for building the calibration curve, Δ*A* values can be converted into a precise concentration of a specific antioxidant whose antioxidant capacity is equivalent to that one of the sample. Typically, Trolox (6-hydroxy-2,5,7,8-tetramethylchroman-2-carboxylic acid) is employed as a standard, and the TEAC (Trolox equivalent antioxidant capacity) value is obtained. In this study, EGCG was employed, allowing for obtaining the EGCG equivalent antioxidant capacity.

The ABTS^•+^ radical was generated by mixing 1 mL of ABTS (2,2′-Azino-bis­(3-ethylbenzothiazoline-6-sulfonic acid) solution in Milli-Q water (7 mM) with 1 mL of potassium persulfate (4.9 mM), followed by incubation in the dark at room temperature for 12–16 h. The ABTS^•+^ radical was then diluted in PBS to reach an initial absorbance near 0.7 at 734 nm. Measurements were performed using a Cary 5000 UV–vis–NIR spectrophotometer (Agilent, Santa Clara, USA). For analysis, 50 μL of each sample or standard was mixed with 950 μL of diluted ABTS in Eppendorf tubes, vortexed for 10 min, and then absorbance at 734 nm was measured. EGCG solutions at 0.025, 0.05, and 0.1 mg/mL concentration were employed as standards; the blank was PBS, and unloaded NPs served as a control.

### 2.4 Cell Culture

Human hepatocellular carcinoma cells (HepG2) and human pancreatic carcinoma cells (PANC-1) were obtained from ATCC (ATCC HB-8065 and ATCC CRL-1469, respectively) Cells were routinely maintained in Dulbecco’s Modified Eagle Medium (DMEM, high glucose) supplemented with 10% (v/v) fetal bovine serum (FBS), 1% penicillin–streptomycin (100 U/mL penicillin, 100 μg/mL streptomycin), and 2 mM l-glutamine. IMDM medium, β-mercaptoethanol, penicillin–streptomycin, l-glutamine, and FBS were purchased from Sigma-Aldrich. Cultures were incubated at 37 °C in a humidified atmosphere containing 5% CO_2_. Cells were subcultured at approximately 70–80% confluence by using 0.25% trypsin–EDTA for detachment. For experiments, HepG2 and PANC-1 cells were seeded at a density of 3 × 10^6^ cells/mL in appropriate culture vessels to ensure logarithmic growth during treatments. The culture medium was replaced every 2–3 days, and only cells between passages 5 and 20 were employed in experimental procedures to guarantee reproducibility.

### 2.5 Cell Viability Assay (MTT)

To evaluate the cytocompatibility and potential cytotoxicity of EGCG-loaded Ch-NPscompared to free EGCG, a WST-8 colorimetric assay was performed using the Cell Counting Kit-8 (CCK-8; cat. no. 96992, Sigma-Aldrich, UK). This assay quantifies cell viability based on the metabolic reduction of the water-soluble tetrazolium salt WST-8 to a soluble formazan dye, which is directly proportional to the number of metabolically active cells. HepG2 and PANC-1 cells were seeded in 96-well plates at a density of 1.0 × 10^4^ cells/cm^2^ in Dulbecco’s modified eagle medium (DMEM) supplemented with 10% fetal bovine serum (FBS), 1% penicillin–streptomycin, and 2 mM l-glutamine. Cells were incubated at 37 °C in a humidified atmosphere with 5% CO_2_. After 24 h to allow for cell attachment, the cells were treated with increasing concentrations of H_2_O_2_ or H_2_O_2_ in combination with EGCG-loaded Ch-NPs. Each experimental condition was tested in triplicate, and untreated wells served as negative controls. After nanoparticle exposure, 10 μL of WST-8 reagent was added to each well, following the manufacturer’s instructions. Plates were incubated for 1 h at 37 °C under standard cell culture conditions. Absorbance was measured at 450 nm using a BioTek Gen5Microplate Reader (Agilent Technologies, USA). The relative cell viability (%) was calculated by normalizing the absorbance of treated samples to that of untreated control cells.

### 2.6 Reactive Oxygen Species (ROS) Production Assay

The intracellular accumulation of ROS was evaluated using the cell-permeant fluorogenic probe 2′,7′-dichlorodihydrofluorescein diacetate (H_2_DCFDA; Sigma-Aldrich). Cells were treated with varying concentrations of H_2_O_2_ alone or pretreated with H_2_O_2_ at different concentrations to induce oxidative damage, followed by exposure to EGCG-loaded Ch-NPs. Fluorescence intensity, reflecting ROS levels, was measured to assess the protective or modulatory effects of the nanoparticles after oxidative stress. Upon diffusion into cells, H_2_DCFDA is enzymatically deacetylated by intracellular esterases into nonfluorescent H_2_DCF, which is rapidly oxidized in the presence of ROS to yield highly ∼fluorescent 2′,7′-dichlorofluorescein (DCF). The cells were seeded in 96-well plates at a density of 10,000 cells/cm^2^ and cultured for 24 h at 37 °C in a humidified 5% CO_2_ atmosphere. After treatment, cells were incubated with 10 μM H_2_DCFDA in serum-free medium for 1 h at 37 °C. Following washing with DPBS (Gibco), fluorescence intensity was measured using a multimode microplate reader (excitation 505 nm, emission 525 nm). Cells treated with 5 mM H_2_O_2_ served as a positive control. Fluorescence values were normalized to untreated controls and expressed as fold-change in intracellular ROS levels.

### 2.7 Confocal Microscopy on PANC-1 and HepG2 Cells Treated with Ch- NPs

To investigate the cellular uptake of EGCG-loaded Ch-NPs, the HepG2 and PANC-1 cells were cultured on glass-bottom dishes (WillCo Wells) and successively exposed to FITC-marked EGCG-loaded Ch-NPs for 6, 24, and 48 h. After incubation, the cells were fixed with 0.25% (v/v) glutaraldehyde in phosphate-buffered saline (PBS) for 10 min at room temperature and subsequently permeabilized with 0.1% (v/v) Triton X-100 in PBS for 3 min. The nuclei were counterstained with DAPI, and plasma membranes were labeled with CellMask Plasma Membrane Stains (Thermo Fisher).

For high-resolution cells’ morphological analyses, HepG2 and PANC-1 cells were even cultured on glass-bottom dishes and exposed either to control NPs or to EGCG-loaded Ch-NPs. To assess the effects of nanoparticle treatment on cellular morphology, cells were first fixed and permeabilized and, successively, stained with 4′,6-diamidino-2-phenylindole (DAPI; Thermo Fisher) for nuclear staining, FITC-conjugated Phalloidin (Thermo Fisher) to mark the F-Actin cytoskeleton, and MitoTracker Deep Red FM (Thermo Fisher) for mitochondrial morphology and localization.

For all the confocal characterizations, the imaging was performed on a Leica TCS SP8 STED 3X super-resolution confocal microscope, equipped with 100×, 63×, and 40× oil-immersion objectives, as well as a 20× dry objective. The system was configured with both photomultiplier tubes (PMTs) and Hybrid (HyD) detectors. Excitation was provided by Argon and HeNe lasers (specific wavelengths indicated for each fluorophore), and acquisition parameters were optimized individually. Images were acquired at a resolution of 1024 × 1024 pixels with a scan rate of 0.6 Hz. The pinhole was set to 1 Airy unit, the offset level to −0.2, and the detector gain was adjusted per channel to maximize signal-to-noise ratio while preventing oversaturation.

### 2.8 Nanoparticles Internalization Evaluation by Amnis ImageStream X Mark II Imaging Flow Cytometer

HepG2 and PANC-1 cells were seeded in 24-well plates at a density of 5 × 10^4^ cells per well and incubated with FITC-conjugated chitosan nanoparticles for 24 h under standard culture conditions (37 °C, 5% CO_2_). After treatment, cells were fixed with 4% paraformaldehyde for 15 min at 37 °C. The cellular internalization and distribution of nanoparticles were analyzed using an Amnis ImageStream X Mark II imaging flow cytometer, (Cytek Biosciences, Inc.; Lakeview Blvd, Fremont, CA 94538), employing different channels: brightfield (Ch0), side scatter (SSC), and fluorescence channels excited at 405 and 488 nm. Cells were acquired at low flow speed using a 60× objective (NA = 0.9, DOF = 2.5 μm, core size = 7 μm) to optimize sensitivity, with standard D-PBS sheath fluid. Nanoparticle internalization was assessed using the Internalization wizard in IDEAS v6.4 software, which quantifies the extent of uptake by calculating the ratio of fluorescence intensity within the cell interior to the total cellular fluorescence. The wizard provides statistical outputs, including cell count and percentage gated for each sample. Internalization efficiency was reported as the percentage of cells exhibiting significant intracellular localization of nanoparticles. Analyses were performed on a minimum of 1000 cells per sample, following standard gating strategies to exclude debris, doublets, and nonviable cells, ensuring accurate quantification of cellular uptake.

### 2.9 Software

Topographical characterization of the EGCG-loaded Ch-NPs was conducted via Atomic Force Microscopy (AFM), with image acquisition and initial data processing carried out using the NOVA_PX software suite (NT-MDT Spectrum Instruments, Moscow, Russia). To correct for common artifacts of the AFM imaging, such as background bowing effect and three-dimensional distortion, advanced image correction routines were applied using the Image Analysis P9 (IA-P9) software, also provided by NT-MDT Spectrum Instruments. These corrections ensured accurate quantification of surface roughness parameters and enhanced the reliability of the topographical data. For detailed morphological analysis of nanostructured features, including NPs dimensions and HepG2 and PANC-1 cells’ morphological parameters (cell size, nucleus shape and dimension, and mitochondrial activity), the open-source software ImageJ (version 1.47v; National Institutes of Health, Bethesda, MD, USA) was employed. The confocal images processing was carried out by means of the Leica application suite X (LasX, Leica Microsystems) software. Finally, the molecule’s sketch representation was carried out by using MolView online free software (available on https://molview.org/).^24^

All experimental data were subsequently processed, plotted, and statistically analyzed using OriginPro software (version 2024b; OriginLab Corporation, Northampton, MA, USA). To ensure visual accessibility and facilitate accurate interpretation across a diverse readership, graphical outputs were generated using color palettes specifically designed to be distinguishable by individuals with color vision deficiencies.^25^ This consideration aligns with current best practices for inclusive scientific data presentation.

### 2.10 Statistics

Statistical analyses were performed using OriginPro software (OriginLab, version 8.5, Northampton, MA, USA). All data were expressed as mean ± standard deviation (SD) from at least three independent experiments. This assay confirmed the general biocompatibility of the nanoparticle formulations and was used to establish appropriate concentrations for subsequent uptake and functional assays. For data analysis, one-way analysis of variance (ANOVA) was applied, followed by Tukey’s HSD test for post hoc comparisons. A significance threshold of *p ≤ 0.05 was used. Unless noted otherwise, all results are expressed as mean ± standard deviation, based on five independent replicates (*n* = 5).

## 3 Results and Discussion

Ionic gelation is a bottom-up approach that offers significant advantages over other chitosan nanoparticle fabrication methods, such as electrospray, emulsification, solvent diffusion, and microemulsion.^26–28^ Unlike these techniques, ionic gelation employs mild, solvent-free conditions without the need for elevated temperatures or high shear forces.^28^ As a result, it is cost-effective, biocompatible, and particularly well-suited for preserving the structural and functional integrity of sensitive bioactive agents. The ionic gelation process (Figure 1) is based on electrostatic interactions between the protonated amino groups of chitosan (R–NH_3_^+^) (Figure 1a) and the negatively charged phosphate groups (−O^–^) of sodium tripolyphosphate (TPP) (Figure 1b) and the negatively charged group (−O^–^) of epigallocatechin gallate (Figure 1c). Upon mixing, these oppositely charged moieties form a chitosan–TPP (Ch/TPP) complex, where ionic cross-linking results in the establishment of a polyelectrolyte network stabilized by multiple Ch–NH_3_^+^/TPP–O^–^ interactions.^11^ The progressive organization of this network gives rise to an entangled three-dimensional matrix, which spontaneously precipitates from aqueous solution as nanosized gel-like particles (Figure 1d). A critical parameter governing this process is the acid–base behavior of the primary amino groups of chitosan (p*K*_a_ ≈ 6). In mildly acidic aqueous media (pH < 6.5), these groups are predominantly protonated (R–NH_2_ + H^+^ → R–NH_3_^+^), thereby increasing the density of available cationic sites for ionic cross-linking with TPP. This pH-responsive characteristic of chitosan underlies the efficiency of nanoparticle formation and enables precise modulation of physicochemical properties through control of the reaction conditions.^11,17,29,30^

However, while most reported CS–TPP systems rely on the passive entrapment of EGCG during nanoparticle formation, our synthesis protocol is based on the precomplexation of EGCG with TPP prior to chitosan addition, thereby actively involving the polyphenol in the nucleation and assembly process. This sequential approach resulted in highly reproducible nanoparticle formation and stable colloidal suspensions under ambient and sterile conditions, addressing common limitations such as low encapsulation efficiency and poor batch-to-batch reproducibility reported for conventional one-step methods.^31^ Importantly, modulation of the EGCG/TPP ratio enabled fine control over cargo incorporation without altering polymer concentration or processing parameters, a feature that is rarely achieved in chitosan-based EGCG nanocarriers. Overall, these results demonstrate that our strategy constitutes a methodological advancement over existing chitosan–EGCG nanoparticle formulations. The Ch-NPs and the ECGC-loaded Ch-NPs were synthesized by the ionic gelation method, as described in depth in Section 2. The method mentioned above permits the efficient encapsulation of EGCG while maintaining nanoparticle stability and uniformity. Bare Ch-NPs and EGCG-loaded Ch-NPs were characterized using different techniques. First, dynamic light scattering (DLS) measurements were acquired to collect information about the hydrodynamic diameter, polydispersity index (PDI), and zeta potential (Table 1).

**Table 1:** Average Size (Expressed in nm) ± Standard Deviation, PDI, and ζ Potential of Bare and EGCG-Loaded Ch-NPs from Bare Ch-NPs and Ch-EGCG-NPs Samples Having Different Weight Ratio (w/w) Chitosan: EGCG in Different Volumes

	CS/EGCG (w/w)	particle size (nm)	PDI	zeta potential (ζ) (mV)
Ch NPs (bare)	-	(203 ± 21.8) nm	(0.4 ± 0.12)	(+20.0 ± 0.9)
Ch-EGCG-NPs (100 μL)	1:0.2	(184 ± 17.1) nm	(0.3 ± 0.11)	(+34.0 ± 2.3)
Ch-EGCG-NPs (250 μL)	1:0.5	(213 ± 30) nm	(0.2 ± 0.10)	(+36.7 ± 1.6)
Ch-EGCG-NPs (350 μL)	1:0.7	(205 ± 6.2) nm	(0.2 ± 0.05)	(+39.8 ± 1.2)
Ch-EGCG-NPs (500 μL)	1:1	(236 ± 6.7) nm	(0.3 ± 0.10)	(+45.9 ± 3.8)

As shown in Table 1, bare Ch-NPs exhibited an average particle size of (203.0 ± 21.8) nm. Upon loading with EGCG (100 μL of a 2 mg/mL solution, corresponding to a chitosan/EGCG weight ratio of 1:0.2), the nanoparticle size slightly decreased to (184.0 ± 17.1) nm. Increasing the EGCG content led to a progressive increase in particle size. Specifically, nanoparticles prepared with 250, 350, and 500 μL of EGCG (corresponding to chitosan/EGCG weight ratios of 1:0.5, 1:0.7, and 1:1, respectively) displayed mean diameters of (213.0 ± 30.0) nm, (205.0 ± 6.2) nm, and (236.0 ± 6.7) nm. Across all formulations, the polydispersity index (PDI) remained in the range of 0.2–0.3, indicating monodisperse systems with narrow and homogeneous size distributions, a key requirement for drug delivery applications. The bare Ch-NPs held a positive ζ potential (+20.0 ± 0.9) mV, due to the abundant carboxyl groups of chitosan. On the other hand, the ζ potential of EGCG-loaded Ch-NPs changed from (+34.0 ± 2.3) mV to (+45.9 ± 3.8) mV.

The morphology of the Ch-NPs was investigated using both AFM and TEM to obtain complementary structural information. AFM was employed to analyze particle topography and surface features under near-physiological conditions, while TEM provided high-resolution imaging of particle shape and internal structure, allowing a comprehensive morphological characterization of the NPs systems. The AFM analysis revealed that both the bare Ch-NPs and those loaded with ECGC retained a predominantly spherical and homogeneous morphology. Quantitative image analysis was performed using the Image Analysis-P9 software to estimate the particle size (Figure 2b,d,f,h,j). In agreement with the size distribution data obtained via DLS, a moderate increase in the average size of the CH-NPs was observed upon EGCG loading, from (152.5 ± 32.4) nm for unloaded NPs to (150.1 ± 40.9) nm, (168.7 ± 44.5) nm, and (170.3 ± 50.9) nm for the NPs synthesized with 100 μL, 250 μL, 350 μL, and 500 μL of EGCG, respectively.

**Figure 2:**
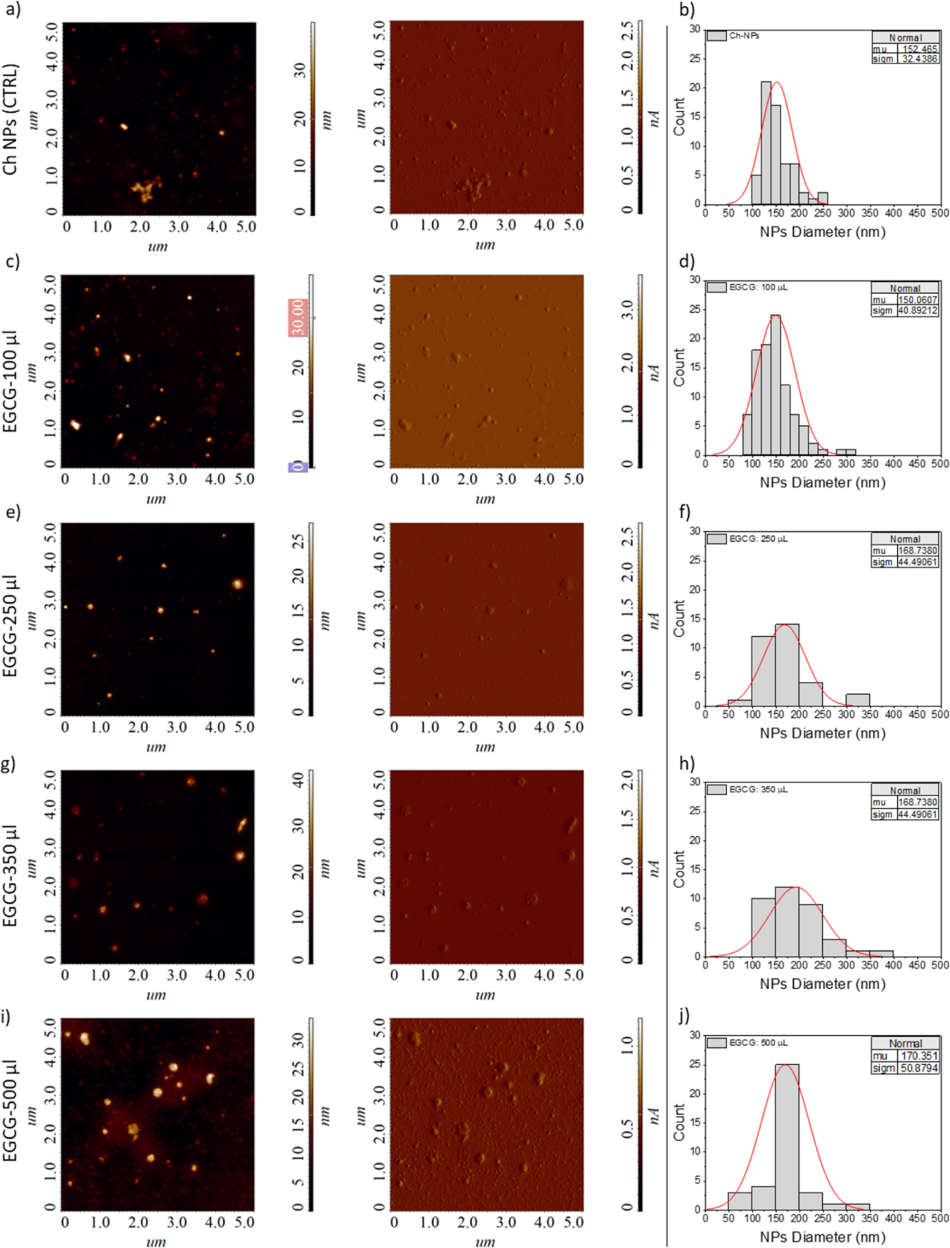
High-resolution AFM characterization, acquired in semicontact error mode on (5 × 5) μm^2^ area, of (a) bare Ch-NPs, and EGCG-loaded Ch-NPs synthesized with (c) 100 μL, (e) 250 μL, (g) 350 μL, and 500 μL of EGCG solution. For each AFM characterization, the SensHeight image is reported on the left side, while the magnitude image is on the right. Particle size distribution, fitted by a Gaussian curve, of (b) bare Ch-NP and EGCG-loaded Ch-NPs synthesized with EGCG (d) 100 μL (weight ratio (w/w) chitosan: EGCG 1:0.2); (f) 250 μL weight ratio (w/w) chitosan: EGCG 1:0.5, (h) 350 μL (weight ratio (w/w) chitosan: EGCG 1:0.7), and (j) 500 μL (weight ratio (w/w) chitosan: EGCG 1:1).

The TEM images provided additional confirmation of the morphological features of both the bare chitosan nanoparticles and those incorporating EGCG (Figure 3). Careful examination of the micrographs indicated that the bare Ch-NPs displayed a relatively uniform spherical morphology with an average diameter of approximately (141.4 ± 31.0) nm (Figure 3a). When the incorporation of EGCG increased, the mean particle diameter increased too, varying from (119.3 ± 12.6) nm, (137.4 ± 25.6) nm, (156.1 ± 14.2) nm, and (167.8 ± 17.0) nm, for the NPs synthesized with 100 μL, 250 μL, 350 μL, and 500 μL of EGCG, respectively (Figure 3b–e). These results were consistent with AFM measurements. The hydrodynamic diameters measured by DLS were consistently larger than those obtained from AFM and TEM, reflecting the different physical states probed by these techniques. Specifically, DLS captures the hydrated size of chitosan nanoparticles in solution, including the polymer-bound water layer, whereas AFM and TEM analyze dehydrated particles; given the polyelectrolytic and hydrogel-like nature of chitosan, such swelling in aqueous environments is expected and accounts for the observed size discrepancies.

**Figure 3:**
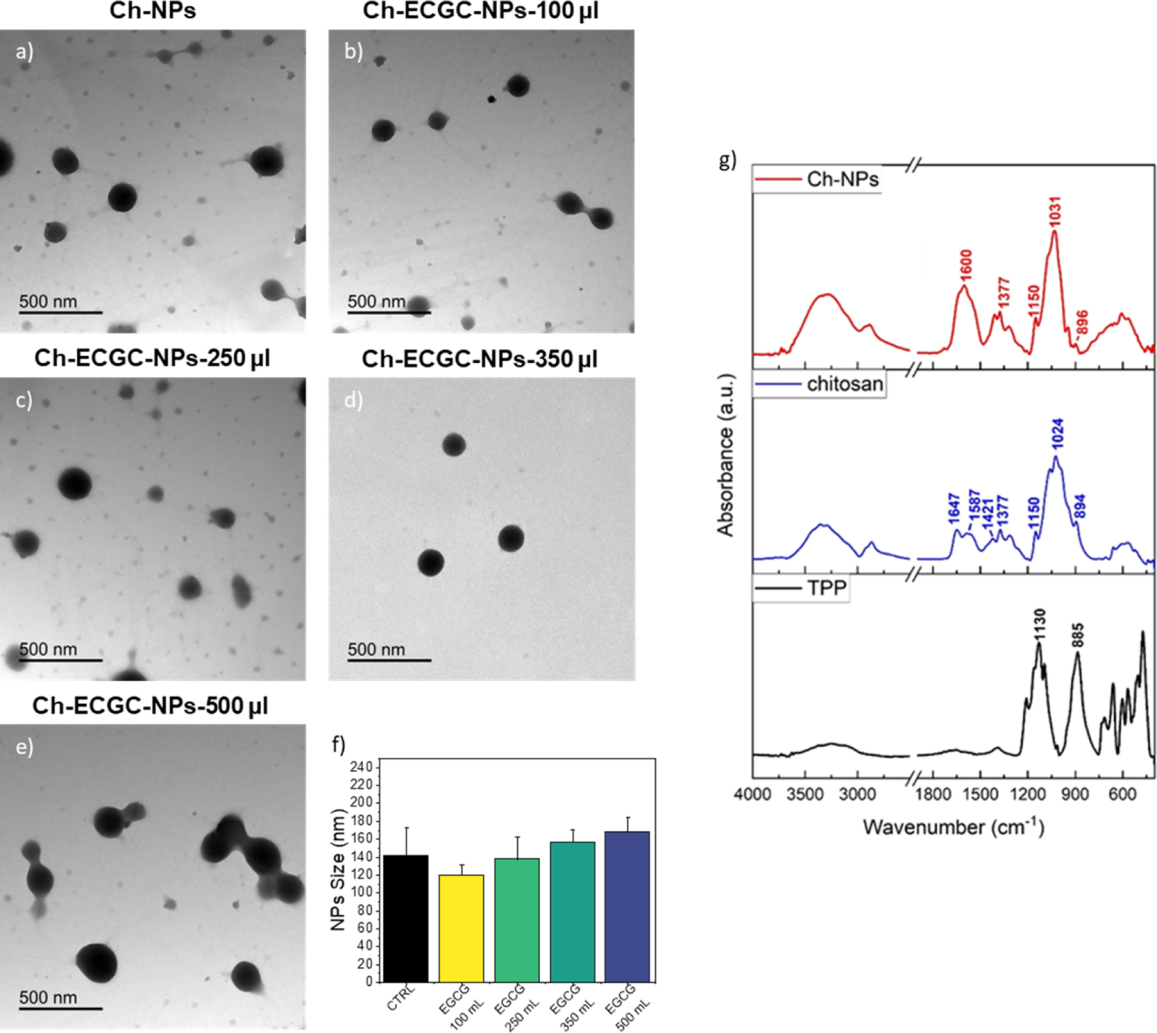
Characterization of Ch-NPs and EGCG-loaded Ch-NPs. Representative TEM images of (a) Ch-NPs (control), (b) Ch-EGCG-NPs–100 μL (weight ratio (w/w) chitosan: EGCG 1:0.2); (c) Ch-EGCG-NPs–250 μL (weight ratio (w/w) chitosan: EGCG 1:0.5), (d) Ch-EGCG-NPs–350 μL (weight ratio (w/w) chitosan: EGCG 1:0.7), and (e) Ch-EGCG-NPs–500 μL (weight ratio (w/w) chitosan: EGCG 1:1). Scale bars: 500 nm. (f) Average NPs size distribution. Data were reported as Mean values ±SD (g) FTIR spectra of Ch-NPs (red line), Chitosan Solution (blue line) and TPP solution (black line).

Figure 3g shows the ATR-FTIR spectra of the cross-linking agent TPP (black trace), chitosan (blue trace), and the Ch-NPs prepared from these two components. It can be observed that the signals corresponding to the stretching vibrations of the phosphorus–oxygen bonds in TPP, the most intense being at 1130 and 885 cm^–1^, were not detectable in the spectrum of the nanoparticles, indicating that the amount of TPP required for NPs formation and incorporated into the particles was relatively low. This can also be explained by the polymeric nature of chitosan and its much higher molecular weight compared to that of TPP. However, the comparison between the spectrum of Ch-NPs and that of the starting chitosan revealed significant changes in molecular vibrations following nanoparticle formation. In the spectrum of the starting chitosan, the Amide I band at 1647 cm^–1^ is well-defined, as is the band at 1377 cm^–1^ (methyl bending), both attributable to residual acetamide groups on the chitosan chains. Additionally, the signals at 1587 and 1421 cm^–1^ were likely due to acetate ions (asymmetric and symmetric stretching of the carboxylate group), which act as counterions. In the Ch-NPs spectrum, a signal at 1600 cm^–1^ emerged more prominently, attributed to protonated amino groups that interact characteristically with the cross-linking agent and the specific chemical environment defined by the NPs structure. The bands associated with C–OH stretching, located between 1200 and 900 cm^–1^, also undergo notable changes upon nanoparticle formation, except for the signal at 1150 cm^–1^, which corresponds to the β-glycosidic bond. Moreover, the spectrum of Ch-NPs showed increased absorbance intensity around 3400 cm^–1^ and 1630 cm^–1^, suggesting a higher water content compared to the starting chitosan. It was likely that water molecules were trapped within the nanoparticle core. Finally, the absorption signals attributed to acetate ions (1587 and 1421 cm^–1^) can be detected in the Ch-NPs spectrum, suggesting that this counterion was retained to some extent in the NPs structure.

The antioxidant capacity of the formulations was assessed using the Trolox Equivalent Antioxidant Capacity (TEAC) assay, expressed as equivalents of EGCG. Free EGCG, used as the positive control, exhibited the highest antioxidant activity (0.92 ± 0.12 mg EGCG equivalents). The EGCG-loaded Ch-NPs were loaded with 500 μL of EGCG solution at 2 mg/mL, corresponding to an initial loading of approximately 1000 μg. The TEAC assay, however, detected an antioxidant capacity equivalent to roughly 500 μg of EGCG (0.49 ± 0.05 mg EGCG equivalents), indicating that a portion of the encapsulated EGCG is not immediately accessible for radical scavenging, likely due to entrapment within the chitosan matrix. Despite this reduction compared to the free molecule, the measurable activity confirms successful EGCG internalization and retention of functional antioxidant capacity within the nanoparticles. In contrast, plain Ch-NPs showed negligible activity (0.02 ± 0.01 mg EGCG equivalents), confirming that chitosan itself does not contribute significantly to radical scavenging and validating its use as a negative control (Table 2) These findings align with previous reports demonstrating that EGCG is the primary contributor to antioxidant activity, while the chitosan carrier mainly provides protection and delivery functions.^32^ Although encapsulation reduces the immediately detectable activity in vitro, it has been shown to enhance stability, protect EGCG from degradation, and allow controlled release, thereby potentially improving its bioavailability and sustaining antioxidant activity in vivo.^33^ Collectively, these results indicate that the nanoparticle system achieves efficient EGCG loading while preserving its bioactivity, supporting its potential application in biomedical contexts where oxidative stress plays a pathogenic role.

**Table 2:** Antioxidant Capacity Determined by TEAC Assay, Expressed as EGCG Equivalents

ID sample	CS/EGCG (w/w)	applied EGCG (mg)	EGCG equivalents in NPs (mg)	ECGC encapsulation yield (%)
Ch-EGCG (100 μL)	1:0.2	0.2	0.09 ± 0.12	35 ± 5.8
Ch-EGCG (250 μL)	1:0.5	0.5	0.23 ± 0.08	42 ± 12.6
Ch-EGCG (350 μL)	1:0.7	0.7	0.32 ± 0.24	43 ± 8.9
Ch-EGCG (500 μL)	1:1	1	0.49 ± 0.05	47 ± 13
Ch-NPs			0.02 ± 0.01	

The cytocompatibility and cytoprotective efficacy of bare Ch-NPs were investigated in human pancreatic carcinoma (PANC-1) and hepatocellular carcinoma (HepG2) cell lines (Figure 4a,b). PANC-1 and HepG2 were chosen as cells models to study EGCG-loaded Ch-NPs because both exhibit oxidative stress but differ in redox capacity and cellular uptake behavior. Their distinct metabolic and endocytic characteristics allow comparison of nanoparticle internalization and antioxidant protection across different biological contexts, enabling evaluation of nanocarrier performance under ROS-induced conditions. Oxidative stress was induced by exposing both the cellular lines to hydrogen peroxide (H_2_O_2_) at varying concentrations for 24 h. Following induction of oxidative stress, cells were post-treated with either free EGCG or EGCG-loaded Ch-NPs for a further 24 h. All experiments were performed using Ch-EGCG (500 μL), corresponding to 0.49 ± 0.05 mg of EGCG and Ch-EGCG (w/w 1:1, chitosan: EGCG). As a control, an equivalent amount of internalized EGCG was administered in its free form.

**Figure 4:**
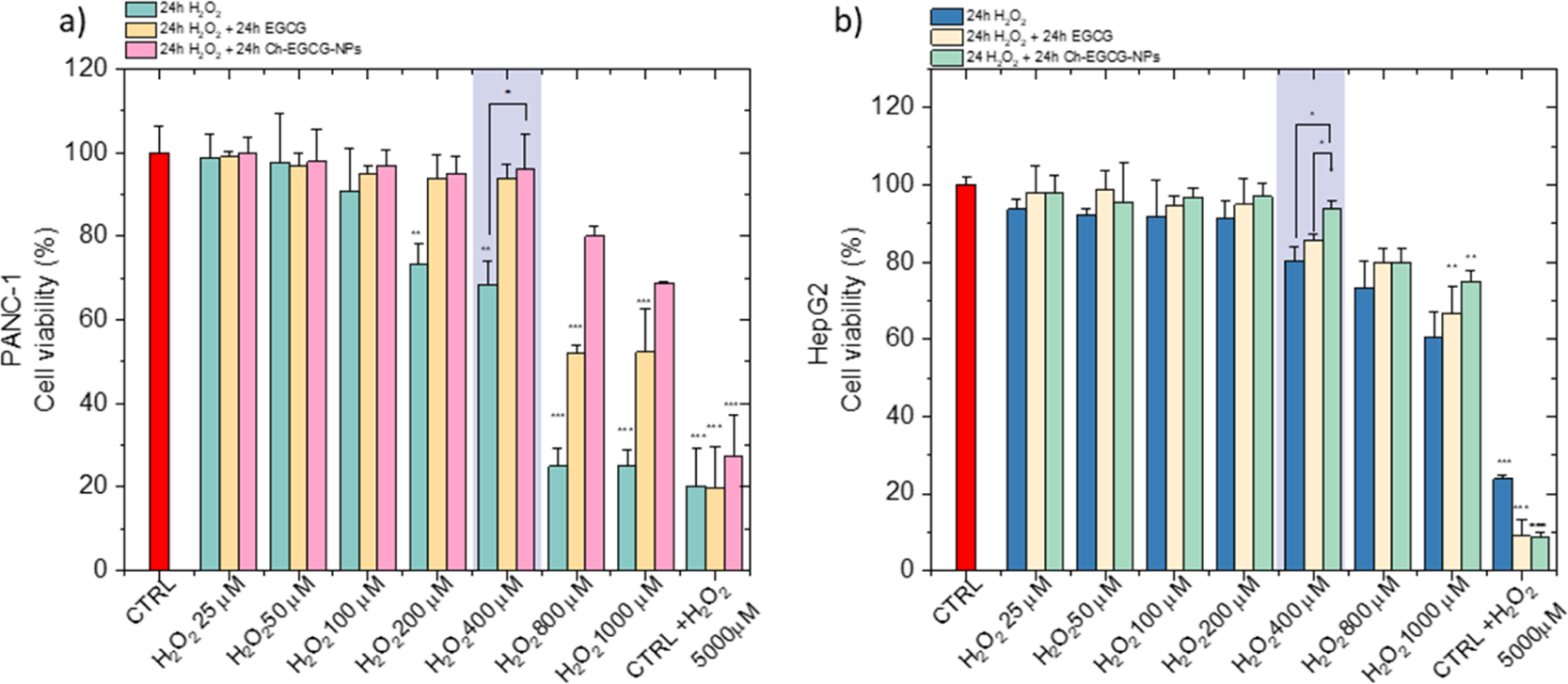
Cell viability of (a) PANC-1 and (b) HepG2 cells assessed by MTT assay. Cells were first exposed to H_2_O_2_ for 24 h to induce oxidative stress and subsequently post-treated with either Ch-EGCG (w/w 1:1, chitosan: EGCG) corresponding to 0.49 ± 0.05 mg of EGCG or free EGCG. Data are presented as mean ± SD (*n* = 3). Statistical significance: *p* < 0.05, *p < 0.01, **p < 0.001 versus untreated control (Red Bars).

Cell viability was quantified at both 24 h post-treatment. The EGCG loaded-Ch-NPs were evaluated for their ability to restore cell viability under oxidative conditions, thus enabling the concurrent assessment of NP safety and therapeutic potential. Specifically, in PANC-1 cells, the exposure to 400 μM H_2_O_2_ for 24 h reduced cell viability by approximately 30%. Under these conditions, free EGCG (0.5 mg/mL) did not significantly modify cell viability, whereas treatment with EGCG-loaded Ch-NPs preserved cell viability at nearly 100% (Figure 4a,b). These findings supported the hypothesis that NP-mediated delivery enhanced the biological efficacy of EGCG in the context of oxidative stress. Similarly, in HepG2 cells, the exposure to H_2_O_2_ for 24 h at high concentrations did not significantly reduce cell viability compared to PANC1, due to their efficient antioxidant defenses and metabolic adaptability.^34^ Under these conditions, free EGCG maintained viability at approximately 85%, while EGCG-Ch-NPs preserved viability close to 100%. Although the difference between treatments was not statistically significant, a trend toward enhanced protection with the nanoparticle system was observed. Similar observations have been reported in other models, where the nanocarrier delivery of EGCG improved stability, cellular uptake, and functional activity compared to the free compound. These results demonstrate that, despite the intrinsic resistance of HepG2 cells to H_2_O_2_-induced oxidative stress, EGCG-loaded Ch-NPs markedly enhanced the preservation of cell viability compared to free EGCG, highlighting the superior protective efficacy of the nanoformulated system (Figure 4a,b). This enhanced effect likely arise from the improved molecular stability, more efficient intracellular delivery, and sustained release provided by the nanocarrier system. In line with previous findings, the protective activity of EGCG-Ch-NPs underscored the ability of nanocarriers to markedly enhance EGCG stability as well as the bioavailability and cellular uptake. In previous works, EGCG-coated gold nanoparticles (E-GNPs) exhibited enhanced anticancer activity compared to free EGCG, attributed to increased stability and reduced cellular efflux.^35^ Recently, EGCG-selenium NPs were developed in order to effectively scavenged ROS and protected PC12 cells from H_2_O_2_-induced damage, confirming the enhanced functional activity of EGCG when delivered via nanocarriers.^36^ Collectively, these findings highlight nanocarrier-based delivery systems as a powerful and efficient strategy to fully exploit the antioxidant and cytoprotective potential of EGCG in PANC-1 cells.

Time-course experiments were performed in HepG2 and PANC-1 cells to characterize H_2_O_2_-induced oxidative stress, The cells were exposed to increasing H_2_O_2_ concentrations for 1, 3, 6, and 24 h, and ROS production was quantified. Both cell lines exhibited a dose-dependent increase in ROS after H_2_O_2_ treatment, with a significant induction observed at 400 μM of H_2_O_2_. More specifically, PANC-1 cells exhibited a delayed ROS accumulation, with a significant increase observed at 6 h, corresponding to an approximately 2.5-fold elevation compared with untreated controls. In contrast, in HepG2 cells, ROS levels increased rapidly, reaching ∼2.0-fold over baseline at 3 h (*p* < 0.05) and declining thereafter. Thus, the two models differ primarily in the kinetics of oxidative stress induction rather than in sensitivity to H_2_O_2_ concentration.

These temporal differences likely reflected variations in intrinsic antioxidant capacity. Indeed, HepG2 cells are known to possess elevated levels of glutathione and catalase, which can buffer ROS accumulation and delay oxidative stress, whereas PANC-1 cells exhibit weaker basal defenses, leading to earlier ROS buildup. Such heterogeneity is consistent with previous reports that redox responses are strongly cell–type–dependent and shaped by both constitutive and inducible defense pathways.^37–39^ After defining the conditions for ROS induction, the protective role of EGCG-loaded Ch-NPs was investigated. In PANC-1 cells, treatment effectively prevented the delayed oxidative burst, lowering the 6 h peak below levels observed in untreated controls. On the other hand, in HepG2 cells, Ch-EGCG-NPs markedly attenuated ROS accumulation, reducing the 3 h oxidative peak near baseline, with sustained suppression at later time points. Together, these findings demonstrate that Ch-EGCG-NPs significantly blunt H_2_O_2_-induced oxidative stress in both HepG2 and PANC-1 cells, albeit with distinct temporal dynamics. In HepG2, the nanoparticles counteract the rapid oxidative burst, whereas in PANC-1, they suppress the delayed response. These results underscore the importance of tailoring oxidative stress models to the intrinsic biology of each cell type and highlight the potential of Ch-EGCG-NPs as versatile antioxidants capable of modulating both early and late phases of ROS accumulation in tumor cells.

To observe cellular internalization and distribution, chitosan nanoparticles were tagged with fluorescein isothiocyanate (FITC). The labeling exploited the reactivity of the FITC isothiocyanate group with the primary amino groups on the chitosan polymer, forming stable thiourea bonds. This approach allows stable fluorophore attachment to the nanoparticle surface without significantly affecting their physicochemical characteristics.^40,41^

First, the fluorescence measurements confirmed the successful conjugation of FITC to the Ch-NPs. As reported in Table 3, the total fluorescence of the initial 5 μM FITC solution was 98,750 au, while the fluorescence detected in the supernatant after nanoparticle purification was 12,740 au According to eq 1, this corresponds to a labeling efficiency of approximately 87%, indicating that the majority of the FITC molecules were covalently bound to the chitosan structures. These results demonstrated an effective conjugation process, ensuring sufficient fluorescence for subsequent imaging and uptake experiments.

**Table 3:** Efficiency of FITC Conjugation to Ch-NPs

ID sample	total fluorescence (a.u.)	supernatant fluorescence (a.u.)	labeling efficiency (%)
5 μM FITC	98750		
Ch-NPs		12740	87%

The cellular internalization of Ch-EGCG-NPs was evaluated in HepG2 and PANC-1 cells using confocal laser scanning microscopy. To visualize intracellular distribution, Ch-EGCG-NPs were fluorescently labeled with FITC and incubated with cells for 6, 24, and 48 h prior to analysis. As shown in Figure 6, FITC-labeled Ch-EGCG-NPs appeared as distinct punctate fluorescent signals dispersed throughout the cytoplasm, confirming effective nanoparticle uptake. No preferential accumulation within specific intracellular compartments was observed, suggesting a predominantly diffuse cytosolic distribution.

**Figure 5:**
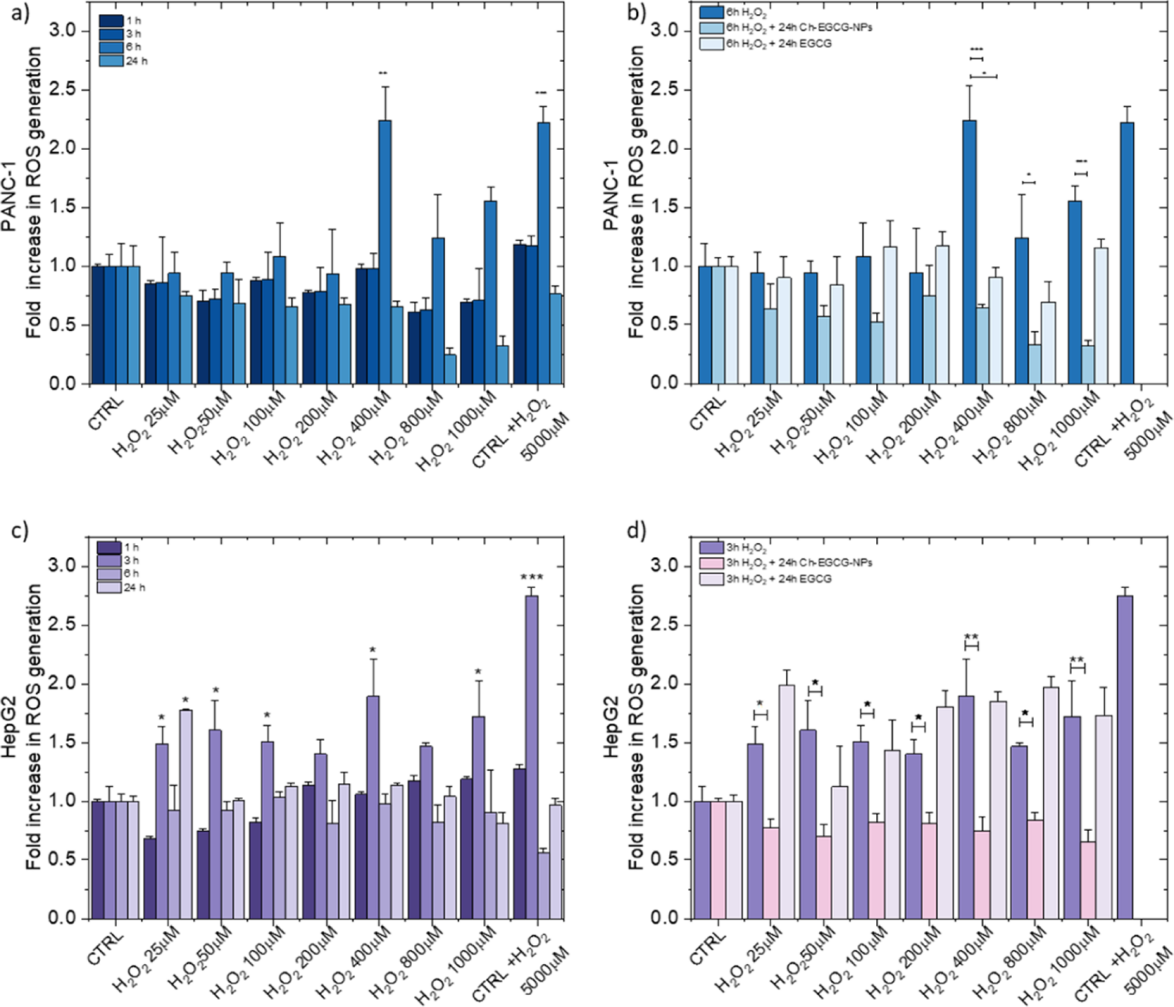
Effect of Ch-EGCG-NPs on ROS generation in HepG2 and PANC-1 cells. (a,c) Time-dependent ROS increase in PANC-1 (a) and HepG2 (c) cells exposed to H_2_O_2_ (1 h, 3 h, 6 h, 24 h). (b,d) ROS levels in PANC-1 (b) and HepG2 (d) cells pretreated with H_2_O_2_ and subsequently exposed to Ch-EGCG (w/w 1:1, chitosan: EGCG) corresponding to 0.49 ± 0.05 mg of EGCG or free EGCG. ROS were quantified by DCFH-DA fluorescence and expressed as fold change relative to untreated controls (mean ± SD, *n* = 3). *p < 0.05, **p < 0.01, ***p < 0.001.

**Figure 6:**
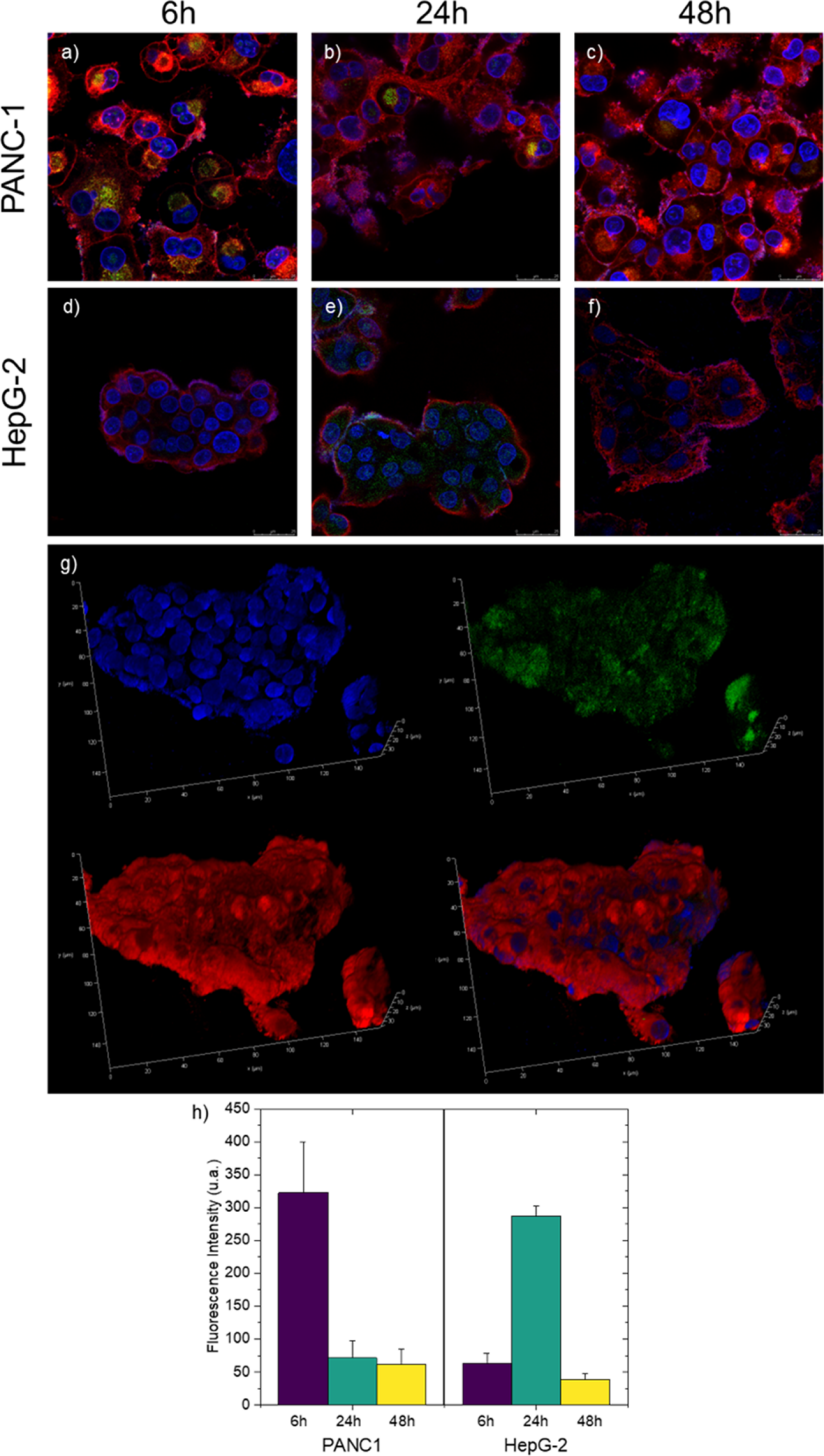
High-resolution confocal fluorescence microscopy images showing the cellular uptake of FITC-labeled EGCG-loaded Ch-NPs by PANC-1 and HepG2 cells. Panels (a–c) show PANC-1 cells after 6, 24, and 48 h of incubation, respectively, while panels (d–f) display HepG2 cells under the same incubation times. Panel (g) represents a 3D confocal reconstruction of a HepG2 cell after 24 h of nanoparticle treatment, illustrating the intracellular distribution of nanoparticles within the cytoplasm. The 3D image was reconstructed from approximately 50 z-stacks. Panel (h) quantifies nanoparticle uptake in PANC-1 and HepG2 cells based on FITC fluorescence intensity measured after 6, 24, and 48 h of incubation. In all images, nuclei are stained with DAPI (blue), and cell membranes are stained with CellMask Plasma Membrane Stain (red).

The uptake of chitosan-based nanoparticles is influenced by several factors, including particle size, surface charge, cell type, and experimental conditions. Generally, nanoparticles smaller than 200 nm are internalized mainly via clathrin-mediated or caveolae/lipid raft–dependent endocytosis,^42^ ^,^ ^43^ whereas larger particles in the micrometre range are more efficiently taken up by phagocytic cells such as macrophages and dendritic cells. Cellular uptake is typically energy-dependent and strongly temperature sensitive. Moreover, positively charged nanoparticles are internalized more efficiently than negatively charged ones in both phagocytic and nonphagocytic cells.^44,45^ Since no single uptake pathway is universally applicable, the internalization mechanisms of chitosan nanoparticles depend on their physicochemical properties as well as the specific cell type involved. In PANC-1 cells, NPs internalization was rapid, with detectable uptake as early as 6 h (Figure 6), characterized by strong green fluorescence distributed throughout the cytoplasm. However, fluorescence intensity progressively declined at 24 and 48 h (Figure 6b–c), indicating of a possible nanoparticle degradation or disassembly. In contrast, HepG2 cells exhibited a delayed uptake profile: fluorescence was weak at 6 h (Figure 6d) but increased substantially by 24 h (Figure 6e) and remained detectable at 48 h (Figure 6f). This observation was further supported by confocal Z-stack three-dimensional imaging (Figure 5g). Comparative analysis reveals faster internalization of Ch-NPs in PANC-1 cells compared to HepG2 cells, whereas nanoparticle retention is more pronounced in HepG2. Quantitative ImageJ analysis of cytosolic fluorescence intensity corroborated these observations (Figure 6h), with PANC-1 cells reaching maximal fluorescence at 6 h and HepG2 cells at 24 h. Overall, these results suggest that the distinct uptake kinetics between the two cell lines likely stem from intrinsic differences in membrane properties or endocytic activity.

In addition to confocal microscopy, cellular uptake was quantitatively assessed using flow cytometry measurements in PANC-1 and HepG2 cells after 6 h and 24 h incubation with FITC-labeled nanoparticles. The analysis showed efficient nanoparticle uptake in both cell lines, with internalized fluorescence detected in approximately 96% of PANC-1 cells and 83% of HepG2 cells. These results indicate predominantly intracellular nanoparticle localization, supporting effective cellular internalization rather than surface association (Figure 7).

A smaller proportion of cells (about 4% in PANC-1 and 10–12% in HepG2) displayed fluorescence restricted to the cell periphery, suggesting incomplete internalization. This could reflect the nanoparticle adhesion to the plasma membrane, incomplete washing during sample preparation, or early stages of endocytic processing prior to full uptake. Importantly, the results obtained by ImageStream were consistent with confocal microscopy imaging, reinforcing the conclusion that most particles were actively internalized into the cytoplasmic compartment. Interestingly, PANC-1 cells displayed a higher percentage of internalization compared with HepG2. Pancreatic cancer cells are characterized by an enhanced capacity for nutrient scavenging and macropinocytosis, which is considered a metabolic adaptation to their nutrient-poor microenvironment.^46,47^ Such increased endocytic activity could account for the higher uptake efficiency observed in PANC-1 compared with hepatocellular carcinoma cells. Furthermore, differences in receptor expression (e.g., CD44 and other scavenger receptors), plasma membrane composition, and cell size or morphology may influence nanoparticle adhesion and entry.^48,49^ Although technical factors such as minor differences in sample handling cannot be completely excluded, the concordance between ImageStream and confocal results suggested that the observed difference largely reflected true biological variation.

These findings are consistent with previous reports demonstrating the robustness of the Amnis internalization tool in distinguishing intracellular from surface-bound fluorescence through erosion masks and spatial redistribution algorithms.^50,51^ Compared with conventional flow cytometry, the image-based confirmation provided by ImageStream reduces false positives associated with surface-associated fluorescence and strengthens confidence in uptake quantification.

The high degree of internalization in both PANC-1 and HepG2 cells indicated that the tested nanoparticles possess favorable physicochemical features, such as size, charge, and surface chemistry, that facilitate efficient cellular entry. These results align with earlier studies reporting strong uptake of chitosan- and lipid-based nanocarriers in epithelial and hepatic tumor models.^52^ Nonetheless, the residual fraction of membrane-associated particles highlights the importance of further optimization, possibly through targeted surface modifications, to maximize receptor-mediated uptake and minimize nonspecific binding.

Then, to evaluate the effect of EGCG and H_2_O_2_ treatments on PANC-1 and HepG2 cell lines, and the successive antioxidant effect of EGCG-Ch-NP treatment, confocal images of those cellular lines were obtained. Cells were treated with hydrogen peroxide at a concentration of 400 μM, with EGCG-loaded Ch-NPs and free EGCG. After 24 h of treatment with H_2_O_2_, the samples were incubated with EGCG-loaded Ch-NPs for another 24 h. Confocal images were analyzed by ImageJ software to quantify variations in cell morphology, mitochondrial fluorescence, and nuclear characteristics (Figures 8 and 9).

**Figure 7:**
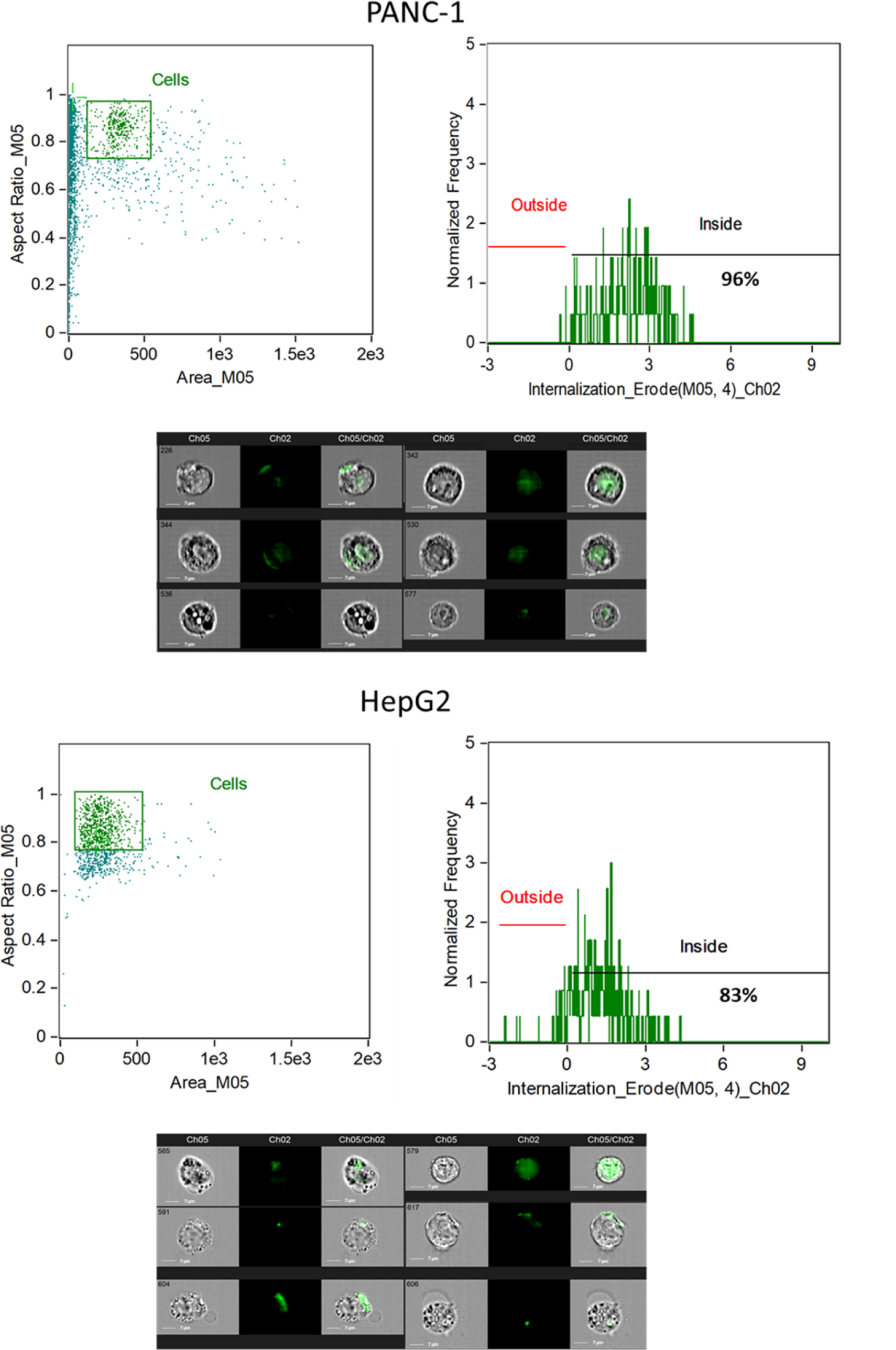
Ch-EGCG NP internalization in HepG2 and PANC-1 cells analyzed by ImageStream. NPs internalization in PANC-1 and HepG2 cells analyzed after 6 h and 24 h post-treatment. Trypsinized cells were analyzed for bright field (Ch05) and Ch-EGCG NPs (Ch02) at 60× magnification. Percentages of focused cells showing Ch-EGCG NPs spots and Internalization Erode median parameters are reported. Ch-EGCG NPs internalization was analyzed using the “Internalization” wizard with the following gating strategy: single cells were gated (Area/Aspect Ratio Intensity, Cells); Cells was then gated for focused cells using the “Gradient-RMS” feature, and Ch02 intensity was measured with the “Internalization” feature applying an “Erode” mask. Focus gate (not shown) identified focused cells. Internalization scores were calculated using the Internalization_Erode feature, with the threshold separating “Inside” versus “Outside” indicated by the horizontal line. Scale bars: 7 μm.

**Figure 8:**
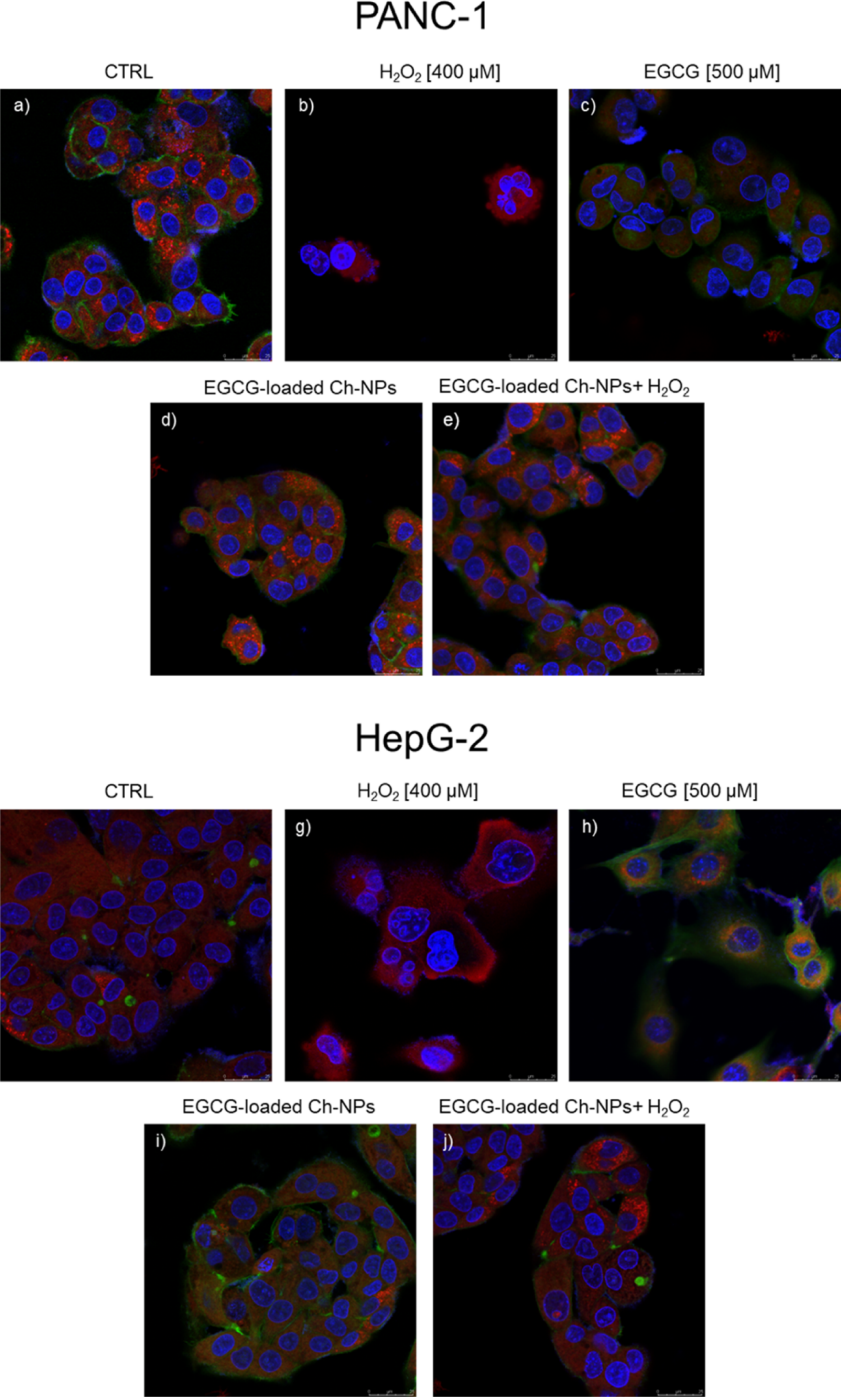
High-resolution confocal fluorescence microscopy images of PANC-1 cells (a) untreated (CTRL), (b) treated with H_2_O_2_ at a concentration of 400 μM, (c) exposed to EGCG 500 μM, (d) incubated with EGCG-loaded Ch-NPs and treated with H_2_O_2_ and successively incubated with EGCG-loaded Ch-NPs of PANC-1 and HepG2, respectively. The cells’ nuclei were stained with DAPI (blue), the mitochondria with MitoTracker Deep Red (red), while the FITC-conjugated phalloidin was used to label the F-Actin cytoskeleton.

**Figure 9:**
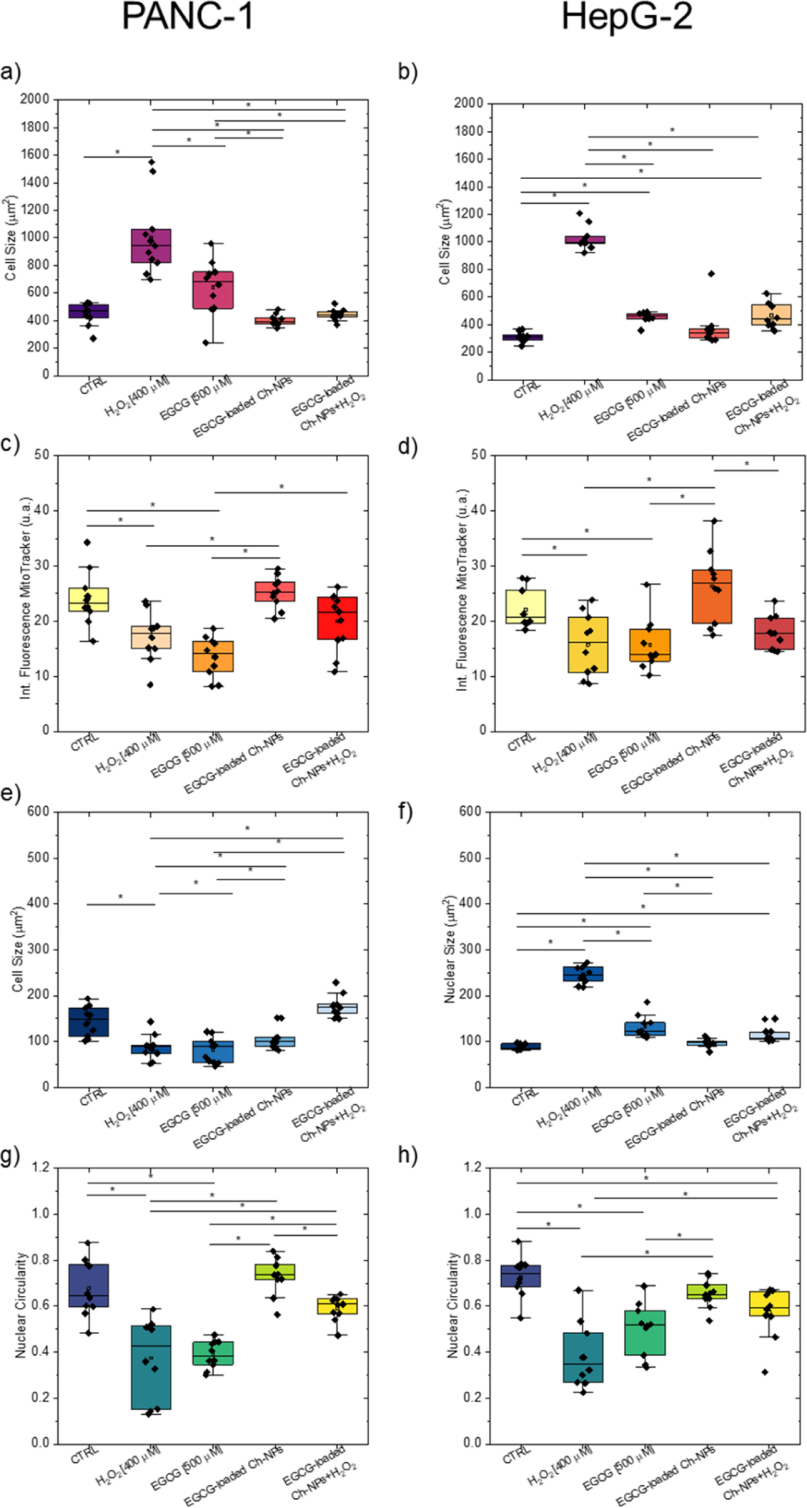
Quantitative morphometric analysis of PANC-1 and HepG2 cells under oxidative stress and treatment with EGCG-loaded Ch-NPs. Analyses were performed using ImageJ software. (a,b) Variation in cell area of PANC-1 and HepG2 cells after exposure to H_2_O_2_, EGCG, and EGCG-loaded Ch-NPs, and after combined treatment with H_2_O_2_ for 24 h followed by Ch-EGCG-NPs for an additional 24 h. (c,d) Mitochondrial fluorescence intensity of PANC-1 and HepG2 cells under the same experimental conditions. (e,f) Nuclear area and (g,h) nuclear circularity of PANC-1 and HepG2 cells following identical treatments. Data are presented as mean ± SD (*n* = 3); *p < 0.05.

As shown in Figure 8(b,g), the treatment with H_2_O_2_ induced drastic variations in cell morphology for both PANC-1 and HepG2 cell lines: the cellular size increased, the shape of the nucleus varied drastically (from circular to crumpled), the number of cells decreased, and the fluorescence of the mitochondria was markedly reduced. Dramatic morphological changes were also observed in cells incubated with free EGCG. In this case, cells appeared separated due to loss of cell–cell contacts, nuclei showed signs of curling, and MitoTracker fluorescence intensity was lower compared to the untreated control, as shown in Figure 8(c,h). The decrease in MitoTracker fluorescence observed after oxidative stress treatment can be attributed to mitochondrial dysfunction induced by ROS accumulation. Because MitoTracker retention depends on mitochondrial membrane potential, loss of fluorescence likely reflects depolarization of the mitochondrial membrane and impaired mitochondrial activity under oxidative conditions,^53^.^54^ These observations are consistent with the known antitumoral properties of Epigallocatechin.^55^ On the other hand, cells incubated with EGCG-loaded Ch-NPs did not exhibit relevant changes in overall cell morphology or nuclear structure, confirming the biocompatibility of chitosan-based nanoparticles (Figure 8d,i).^56^ Finally, cells treated for 24 h with EGCG-loaded Ch-NPs after H_2_O_2_ exposure displayed only mild alterations in their morphology, more closely resembling untreated controls than the H_2_O_2_ or EGCG-treated groups, indicating that EGCG-loaded Ch-NPs effectively rescued cellular architecture (Figure 8e,j).

To corroborate these observations, quantitative analysis using ImageJ was performed (Figure 9). Cell size measurements revealed a significant increase following H_2_O_2_ exposure in both PANC-1 and HepG2 cells. The average cell area increased from (446.9 ± 80.7) μm^2^ to (1003.4 ± 277.8) μm^2^ for PANC-1 and from (310.8 ± 35.3) μm^2^ to (1028.0 ± 87.0) μm^2^ for HepG2, in control and H_2_O_2_-treated conditions, respectively. Treatment with EGCG-loaded Ch-NPsdid not significantly affect cell size in either cell line, showing values comparable to controls. In contrast, free EGCG combined with H_2_O_2_ led to a statistically significant increase in HepG2 cell size compared to controls, whereas no such effect was observed in PANC-1 cells (Figure 9a,b).

The analysis of the mitochondrial fluorescence intensity followed a consistent trend across both cell lines. More in detail, the exposure to H_2_O_2_ resulted in a marked reduction in mitochondrial fluorescence (approximately 28% decrease in both PANC-1 and HepG2) relative to controls. On the other hand, a more pronounced decrease, equal to about ∼43%, was observed in PANC-1 cells treated with free EGCG, compared to a ∼30% reduction in HepG2 cells under the same condition. Conversely, cells treated with EGCG-loaded Ch-NPs showed no statistically significant changes in mitochondrial fluorescence intensity relative to untreated controls. These experimental findings could indicate that the nanoparticles neither induced cytotoxicity, while mitigating the adverse effects associated with the ROS-induced oxidative stress (Figure 9c,d).

In addition, these observations were consistent with the viability assays reported in previous sections. Notably, cells exposed to H_2_O_2_ and subsequently treated with EGCG-loaded Ch-NPs for 24 h partially recovered mitochondrial fluorescence intensity. This finding suggests that treatment with EGCG-loaded Ch-NPs may promote recovery from hydrogen peroxide–induced mitochondrial damage (Figure 9c,d).

The analysis of the nuclear area revealed a shrinkage of ∼40% in the PANC-1 cell line after H_2_O_2_ exposure, consistent with nuclear condensation and contraction, while the enlargement of about 180% of the nuclear size was observed in HepG2 cells. A similar trend, but with a milder variation in the nuclei area, was observed after the EGCG treatment and EGCG-loaded Ch-NPs for both cell lines. On the other hand, the nuclear size tends to return to a size comparable to CTRL after the H_2_O_2_ and is successively treated with EGCG-loaded Ch-NPs (Figure 9e,f).

Finally, nuclear alterations, in terms of circularity, were measured by analyzing the confocal images. The nuclear circularity is one such parameter and is a measure of how close a cell nucleus is to a perfect circle: a circularity of 1 indicates a perfect circle, whereas a circularity of 0 is a substantially elongated shape.^57,58^ The nuclear circularity decreased significantly upon H_2_O_2_ and free EGCG treatments, reflecting abnormal crumpled morphology of the nuclei of both PANC1 and HpeG2 cells. On the other hand, EGCG-loaded Ch-NPs did not affect the cellular circularity values, resulting in comparable values to those of the untreated controls (Figure 9g,h).

Taken together, the qualitative confocal observations and quantitative ImageJ analysis confirm that oxidative stress induced by H_2_O_2_ profoundly alters cell morphology, mitochondrial integrity, and nuclear structure in both PANC-1 and HepG2 cells. Importantly, EGCG-loaded Ch-NPs were more effective than free EGCG in restoring normal cell morphology, nuclear shape, and mitochondrial fluorescence, underscoring the superior antioxidant and protective effects of the nanoparticle-based delivery system.

## 4 Conclusion

Here, we report a rationally engineered nanotherapeutic platform that decisively overcomes the long-standing limitations of EGCG, namely its instability, rapid degradation, and poor intracellular bioavailability. By leveraging ionic gelation, we synthesized EGCG–Ch-NPs with precisely controlled nanoscale dimensions, narrow size distribution, and optimized surface charge, resulting in stable colloidal systems and efficient cellular uptake in both HepG2 and PANC-1 models. Importantly, the nanoparticle formulations were comprehensively, in-depth characterized by complementary physicochemical and structural techniques, and successful EGCG incorporation. The latter not only preserved the intrinsic antioxidant functionality of EGCG but also significantly improved its biological performance. Indeed, compared to the free EGCG molecule, EGCG-Ch-NPs provided a markedly enhanced protection against H_2_O_2_-induced oxidative stress, leading to reduced intracellular ROS accumulation and a significant enhancement of cell viability in both PANC-1 and HepG2 models. Importantly, despite the intrinsic redox dynamics observed between HepG2 and PANC-1 cells, nanoparticle-mediated delivery consistently mitigated oxidative damage, suggesting broad efficacy across biologically divergent cancer phenotypes. High-resolution confocal imaging combined with quantitative morphometric analysis revealed that EGCG-Ch-NPs effectively prevented H_2_O_2_-induced mitochondrial dysfunction, nuclear disorganization, and structural collapse. While free EGCG exhibited partial protection accompanied by concentration-dependent cytotoxicity, nanoencapsulation eliminated these liabilities, preserving cellular integrity under basal conditions and restoring architecture following oxidative insult. These findings demonstrate that nanoparticles do not merely protect EGCG, but the polymer nanostructures also redefine the therapeutic window of the compound. Overall, these findings indicate that EGCG-Ch–NPs system could represent a next-generation redox-modulating nanoplatform, integrating physicochemical stabilization, enhanced intracellular trafficking, and potent functional rescue of oxidative damage. By substantially outperforming the native compound in stability, bioactivity, and cytoprotective efficacy, this strategy establishes a compelling framework for redox-targeted nanomedicine in oxidative stress–driven cancers and paves the way for translational development.

## List of Abbreviations

ABTS: 2,2′-azino-bis (3-ethylbenzothiazoline-6-sulfonic acid)
AFM: atomic force microscopy
ATR: attenuated total reflectance
CCK-8: cell counting kit-8
Ch: chitosan
Ch-NPs: chitosan nanoparticles
EGCG-loaded Ch-NPs: epigallocatechin gallate–loaded chitosan nanoparticles
DAPI: 4′,6-diamidino-2-phenylindole
DCF: 2′,7′-dichlorofluorescein
DCFH-DA/H_2_DCFDA: 2′,7′-dichlorodihydrofluorescein diacetate
DLS: dynamic light scattering
EC: epicatechin
ECG: epicatechin-3-gallate
EGC: epigallocatechin
EGCG: epigallocatechin gallate
FITC: fluorescein isothiocyanate
FTIR: Fourier transform infrared spectroscopy
MTT: (3-(4,5-dimethylthiazol-2-yl)-2,5-diphenyltetrazolium bromide) assay
NPs: nanoparticles
PBS: phosphate-buffered saline
PDI: polydispersity index
ROS: reactive oxygen species
STED: stimulated emission depletion
TEAC: trolox equivalent antioxidant capacity
TEM: transmission electron microscopy
TPP: sodium tripolyphosphate
WST-8: water-soluble tetrazolium salt-8
